# Refractory hypoxaemia in the presence of two common conditions: a case report

**DOI:** 10.1093/ehjcr/ytaf327

**Published:** 2025-07-15

**Authors:** José Gregorio Soto Rojas, Elvira Carrión Ríos, Isabel Maria Jorquera Lozano, Raul Reyes Parrilla, Ricardo Fajardo Molina

**Affiliations:** Department of Cardiology, Hospital Universitario Torrecárdenas, Calle Hermandad de Donantes de Sangre, Almería 04009, Spain; Department of Cardiology, Hospital Universitario Torrecárdenas, Calle Hermandad de Donantes de Sangre, Almería 04009, Spain; Image Department, Hospital Universitario Torrecárdenas, Calle Hermandad de Donantes de Sangre, Almería 04009, Spain; Department of Cardiology, Hospital Universitario Poniente, Carretera de Almerimar, 31, El Ejido, Almería 04700, Spain; Image Department, Hospital Universitario Poniente, Carretera de Almerimar, 31, El Ejido, Almería 04700, Spain; Department of Cardiology, Hospital Universitario Torrecárdenas, Calle Hermandad de Donantes de Sangre, Almería 04009, Spain; Image Department, Hospital Universitario Torrecárdenas, Calle Hermandad de Donantes de Sangre, Almería 04009, Spain; Department of Cardiology, Hospital Universitario Torrecárdenas, Calle Hermandad de Donantes de Sangre, Almería 04009, Spain

**Keywords:** Tricuspid regurgitation, Pulmonary hypertension, Patent foramen ovale, Pacemaker lead, Shunt, Case report

## Abstract

**Background:**

A 72-year-old woman with a history of bicameral pacemaker implantation 12 years earlier due to high-degree atrioventricular block presented with progressively worsening exertional dyspnoea, which recently became evident even with minimal exertion.

**Case summary:**

When the patient consulted, marked hypoxaemia was observed, leading to the exclusion of pulmonary aetiology using computed tomography (CT) angiography. Subsequently, an echocardiogram revealed significant tricuspid regurgitation caused by interference with the pacemaker lead. A bubble study demonstrated a right-to-left shunt in the context of a patent foramen ovale. Given the patient’s age, the initial hypothesis was that the shunt direction resulted from a condition causing severe pulmonary hypertension, resembling Eisenmenger syndrome. However, right heart catheterization ruled out significant pulmonary hypertension. Since the presence of a patent foramen ovale had been documented, we also tested positional changes without observing any improvement in the degree of hypoxaemia, thereby ruling out platypnoea–orthodeoxia syndrome.

**Discussion:**

It was proposed that the direction of the jet and the increase in systolic pressure in the right atrium could explain the type of shunt in the absence of pulmonary hypertension. This was drawing from the nature of pacemaker-mediated tricuspid regurgitation and its rapid progression and eccentricity of the jet. Based on this hypothesis, a percutaneous closure of the foramen was performed, resulting in an almost immediate resolution of the hypoxaemia, as observed in the cath lab.

Learning points

- Tricuspid regurgitation is not an innocuous condition. In a patient with tricuspid regurgitation and an eccentric jet (as commonly occurs in cases of pacemaker lead interference), the clinical presentation with refractory hypoxaemia should prompt consideration of a right-to-left shunt.
- Percutaneous closure of the foramen, which is safe in the absence of significant pulmonary hypertension, effectively resolves the clinical condition in these patients.

## Introduction

The presence of a patent foramen ovale (PFO) is generally regarded as a normal anatomical variant, given its high prevalence in the general population.^1^ Similarly, tricuspid regurgitation (TR) caused by pacemaker lead interference has been a highly prevalent condition that has, until recently, been underestimated in terms of its clinical impact and prognosis.^2^ We present a case in which the interaction between these two conditions leads to the development of a clinically significant right-to-left shunt, manifesting as severe and refractory hypoxaemia.

## Summary figure

**Figure:**
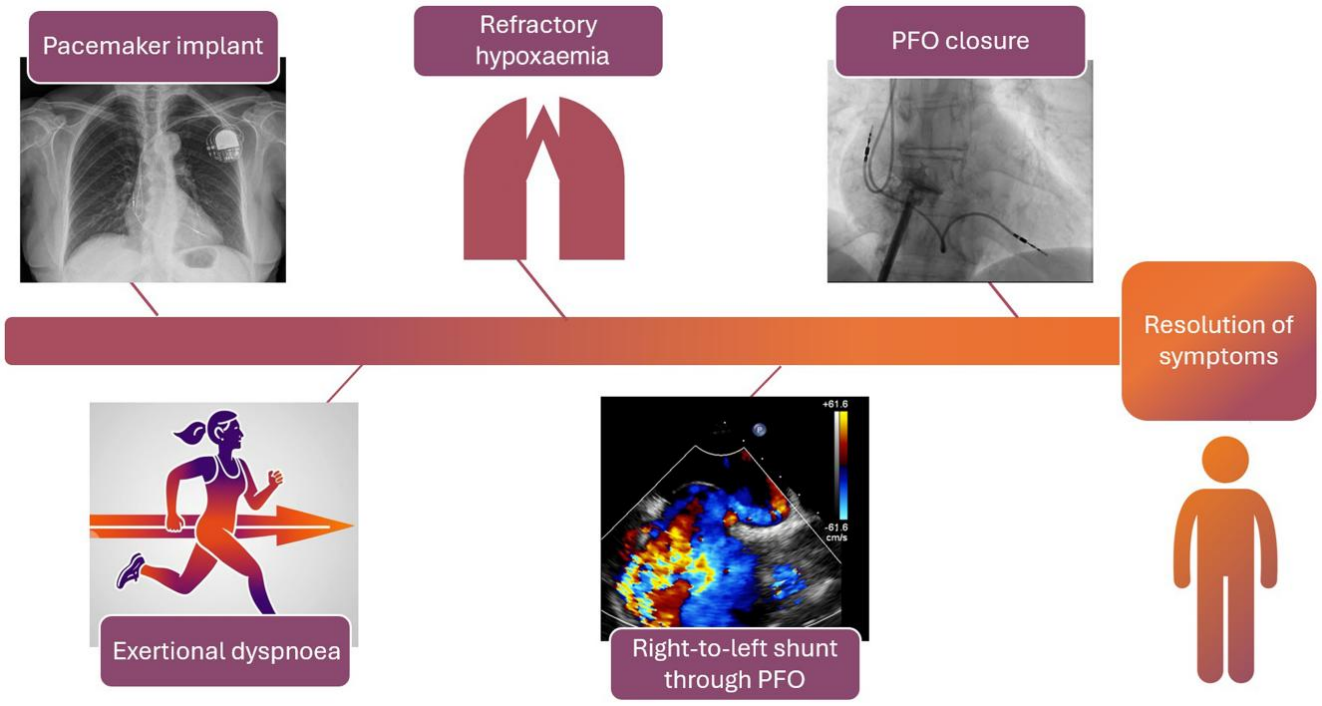
Timeline summarizing the clinical course of a patient with a history of pacemaker implantation who developed a right-to-left shunt through a patent foramen ovale, secondary to tricuspid regurgitation caused by the device lead. Resolution of hypoxaemia was achieved following patent foramen ovale closure. PFO, patent foramen ovale.

## Case presentation

A 72-year-old Caucasian female patient with a history of hypertension and dyslipidaemia, well managed on angiotensin receptor blockers and statins, presented to her primary care physician with a 2- to 3-month history of exertional dyspnoea that progressively limited her daily activities. Her medical history included a dual-chamber pacemaker implanted 12 years ago at another institution for high-grade paroxysmal atrioventricular block, with no further follow-up or echocardiographic data available since the implantation. She reported no orthopnoea or symptoms suggestive of decompensated heart failure, and no other significant comorbid conditions were identified. On examination, breath sounds were normal with no signs of congestion or peripheral oedema. A Grade III/VI systolic murmur was audible at the left sternal border.

The patient was referred to cardiology, where an initial transthoracic echocardiogram (TTE) revealed preserved left ventricular ejection fraction with significant TR due to interference from the pacemaker lead. No other significant abnormalities were identified. Laboratory tests, including N-terminal pro-B-type natriuretic peptide levels, were unremarkable, leading to a referral to pulmonology for further evaluation.

Pulmonary function tests did not reveal any abnormalities. However, baseline pulse oximetry demonstrated an oxygen saturation of ∼85%, prompting referral to the emergency department to exclude acute pathology. A computed tomography pulmonary angiogram ruled out pulmonary embolism and revealed no evidence of interstitial lung disease. Arterial blood gas analysis showed hypoxaemic respiratory failure with associated hypocapnia, necessitating hospital admission for further evaluation.

**Figure 1:**
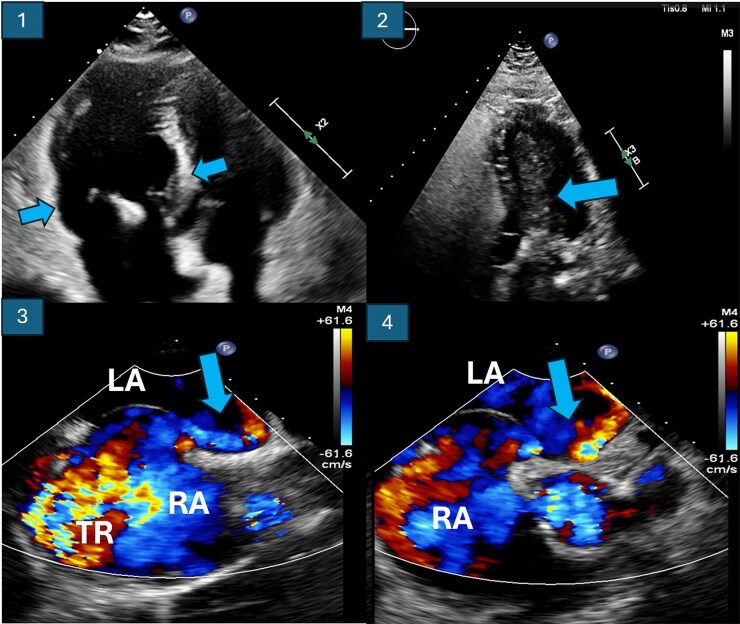
Image 1: Baseline transoesophageal echocardiogram showing right chamber dilation. Image 2: Transthoracic echocardiogram with bubble test demonstrating early massive passage of bubbles into the left chambers. Images 3 and 4: Transoesophageal echocardiogram showing the right-to-left shunt through the patent foramen ovale, as well as the tricuspid regurgitation jet. LA, left atrium; LV, left ventricle; RA, right atrium; RV, right ventricle; TR, tricuspid regurgitation.

The patient required high-flow oxygen therapy, yet her oxygen saturation remained below 90%. Given the severe hypoxaemia refractory to oxygen supplementation, a cardiology consultation was requested during hospitalization to investigate a potential shunt. Repeat TTE revealed persistent moderate-to-severe TR due to pacemaker lead interference, as well as mild right ventricular dilation. A contrast-enhanced bubble study with agitated saline suggested a right-to-left intracardiac shunt, likely through a PFO (*Figure 1*).

Subsequent transoesophageal echocardiography (TEE) confirmed a PFO with a baseline right-to-left shunt, severe TR (in contrast to the moderate-to-severe TR observed on the initial TTE), and findings suggestive of pulmonary hypertension (PH), including a tricuspid regurgitant velocity of 2.8 m/s and right heart chamber dilation (*Figure 1*). Right heart catheterization measured a pulmonary artery systolic pressure of 31 mmHg, mean pressure of 18 mmHg, and a pulmonary vascular resistance of 2.3 Wood units, excluding significant PH.

**Figure 2:**
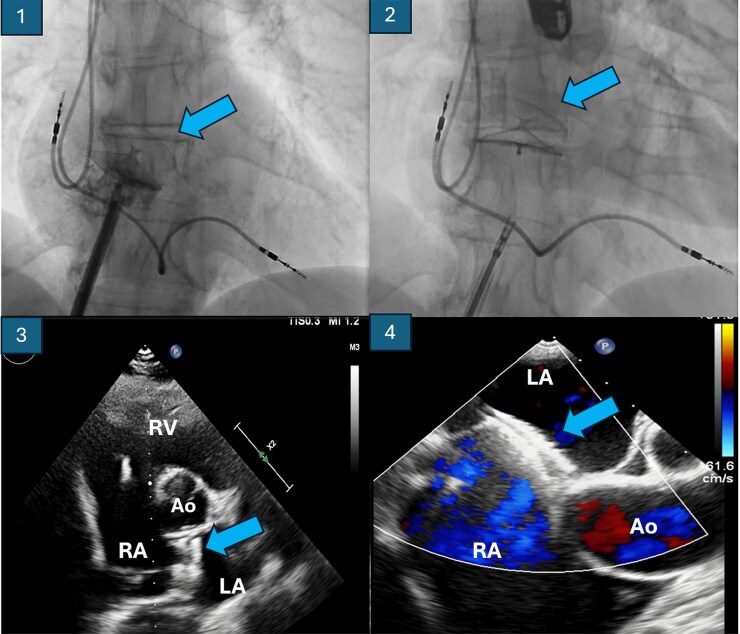
Closure device for foramen. In the images above (1 and 2), fluoroscopy shows the deployment of the device. In the images below, the outcome of the implant is visualized through transthoracic echocardiography on the left (3) and transoesophageal echocardiography (4), without evidence of residual shunt. Ao, aorta; LA, left atrium; RA, right atrium; RV, right ventricle.

Based on these findings, we proceeded with percutaneous closure of the PFO under echocardiographic and angiographic guidance using a Figulla Flex II Uni 28 device via right femoral access (*Figure 2*). The intervention resulted in prompt improvement in arterial oxygen saturation, enabling discontinuation of high-flow oxygen therapy. Post-procedural imaging confirmed proper device placement (*Figure 2*), and the patient was discharged without supplementary oxygen.

The patient continued follow-up at another centre, where she progressively developed symptoms of right heart failure related to the progression of the severe TR. She underwent elective surgery for lead extraction, implantation of a leadless device, and tricuspid annuloplasty. Currently, 2 years after the episode, the patient remains asymptomatic in functional Class I, without further episodes of respiratory failure.

## Discussion

We encountered a patient with refractory hypoxaemia, who had a history of permanent pacemaker implantation and significant TR. A bubble study revealed evidence of a right-to-left shunt, which was subsequently confirmed by TEE, in the presence of a PFO. Contrary to initial suspicion, right heart catheterization ruled out Eisenmenger syndrome by excluding significant elevations in pulmonary arterial pressures.

Although right-to-left shunting through a PFO in the absence of PH has been described,^3^ particularly in the context of platypnoea–orthodeoxia syndrome,^4,5^ our patient did not exhibit postural changes in oxygen saturation, making this diagnosis less likely. Isolated cases have been reported in which a PFO shunt was driven by the direction of the TR jet.^6^ Similarly, in, our case, pacemaker lead-induced TR was identified as a potential contributor to the shunt pathophysiology.

**Figure 3:**
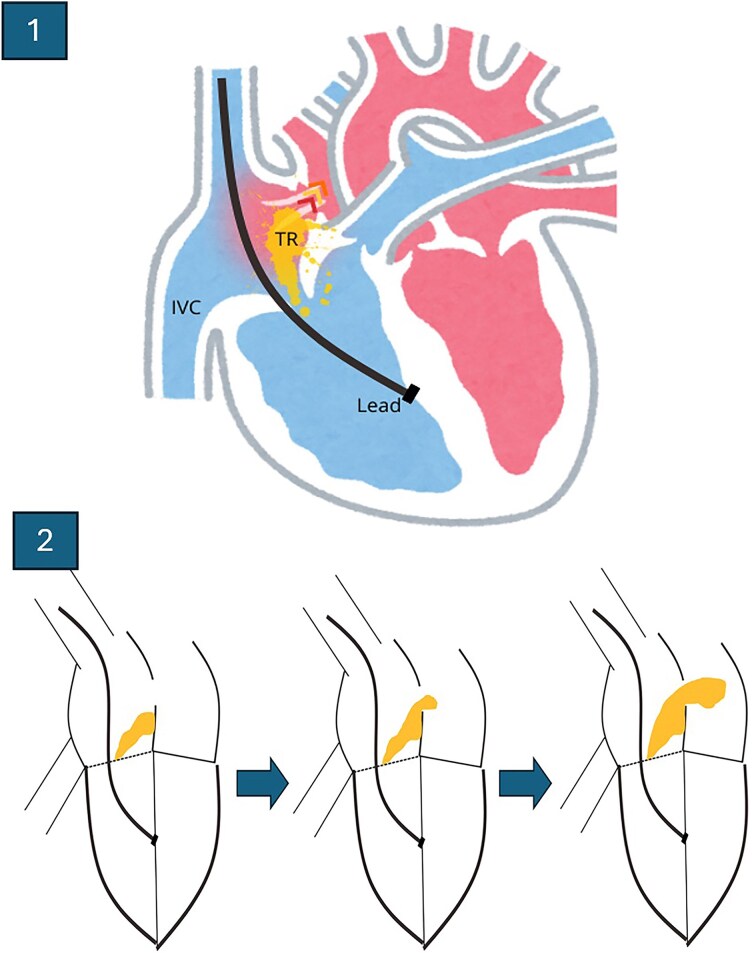
Image 1: Diagram of how pacemaker lead-mediated tricuspid regurgitation generates an eccentric jet directed towards the foramen ovale. Image 2: Schematic evolution of the progression of tricuspid regurgitation and the increase in inflow through the foramen as the severity of regurgitation worsens. IVC, inferior vena cava; TR, tricuspid regurgitation.

It is well known that TR following pacemaker lead implantation is highly prevalent and associated with worse clinical outcomes, with significant regurgitation often developing within the first 2 years post-implantation.^2,7^ Although we lacked serial echocardiographic data from follow-up, the clinical course suggested progressive TR worsening. An eccentric regurgitant jet directed towards the interatrial septum, along with increasing right atrial systolic pressure, likely promoted a right-to-left shunt across the PFO (*Figure 3*). Moreover, the prompt resolution of hypoxaemia after PFO closure reinforces the notion that the foramen, in association with TR, is the underlying mechanism of the clinical presentation.

## Conclusions

In clinical practice, the presence of a PFO is frequently encountered. Notably, a significant proportion of these patients, for diverse clinical indications, will eventually require permanent pacemaker implantation. A well-recognized but often underappreciated complication of this procedure is pacemaker-mediated TR, a condition that remains insufficiently addressed despite its high prevalence. Besides, when TR is attributed to pacemaker lead interference, it tends to progress rapidly, often presenting with eccentric regurgitant jets and portending adverse clinical outcomes.

Contemporary understanding of device-induced TR has evolved. However, many affected individuals continue to lack structured follow-up or targeted management strategies. This case highlights the importance of diagnosing and monitoring pacemaker-mediated TR. Furthermore, it illustrates how the interaction between two common pathologies can lead to severe hypoxaemia in an elderly patient without additional comorbidities.

## Data Availability

All relevant data are included in the manuscript.

## Lead author biography

Dr José Gregorio Soto Rojas is a cardiologist trained at the Torrecárdenas University Hospital. He studied medicine at the University of Seville and currently practices at the Torrecárdenas University Hospital (Almería, Spain).
